# Supplementary material for: “There’s something inside”: Children’s intuitions about animate agents
Source: PLoS One. 2021 May 19;16(5):e0251081. doi: 10.1371/journal.pone.0251081 (PMC8133430; doi:10.1371/journal.pone.0251081)
Supplement: Attachment — Submitted filename: R2R.docx [file pone.0251081.s001.docx]

*Dear Dr. Kominsky,*

*Thank you for submitting your manuscript "There's something inside": Children's intuitions about animate agents” for review and consideration for publication in PLOS ONE. I have now received 3 reviews of the manuscript, and I have carefully read the manuscript myself. The reviews are appended to this email.*

*The Reviewers and I agree that you are addressing an interesting and important question about pre-schoolers’ predictions regarding the insides of self-propelled objects. At the same time, they all raised some concerns about the conclusions you drew based on the present data. Specifically, the main issue, which was mentioned by both Reviewer 1 and Reviewer 2 is that the stimuli you were using for the main experiment could be well considered by the children as mechanical toys, rather than animals. Therefore, considering that at this age probably they already have some experience with self-propelled toys, their expectations regarding the inside of the present objects may not be informative about their biological understanding of animals. I agree with this concern of the reviewers and I am afraid that indeed this could be a serious limitation of your study. Therefore I think unless you can provide sufficient evidence that the subjects you tested perceived the fur-covered self-propelled objects as real animals, you should reformulate the conclusions of your study.*

We have expanded the discussion to consider the issue of mechanical toys and, more generally, the difference between animal-like entities and artificial entities. As such our interpretation of the results is much more cautious.

*As you will see, to deal with this concern, Reviewer 1 proposed to run the same experiment with adult subjects, whereas Reviewer 2 suggested to use stimuli of more realistic animate objects. While I think both of these suggested additional experiments could be informative in relation to your study, I am afraid that they still cannot guarantee that you could hold the same conclusions you have now. Therefore, in light of all these issues, while I am inviting you to submit a revised version of your paper, I would suggest you to prepare your revision after reconsidering your main hypothesis and the potential conclusions you would be able to make based on the present experiment (and on any additional experiments you might plan to run). Also, in case you decide to resubmit a revised version of your paper, please address also all the additional comments of the reviewers.*

We concur that those experiments alone would be inadequate to draw firm conclusions, and so we endeavor to explicitly lay out the central questions and approaches for future research that could more fully address these issues.

*Please submit your revised manuscript in 60 days. If you will need more time than this to complete your revisions, please reply to this message or contact the journal office at plosone@plos.org. When you're ready to submit your revision, log on to* [*https://www.editorialmanager.com/pone/*](https://www.editorialmanager.com/pone/) *and select the 'Submissions Needing Revision' folder to locate your manuscript file.*

*Please include the following items when submitting your revised manuscript:*

- *A rebuttal letter that responds to each point raised by the academic editor and reviewer(s). You should upload this letter as a separate file labeled 'Response to Reviewers'.*
- *A marked-up copy of your manuscript that highlights changes made to the original version. You should upload this as a separate file labeled 'Revised Manuscript with Track Changes'.*
- *An unmarked version of your revised paper without tracked changes. You should upload this as a separate file labeled 'Manuscript'.*

*If you would like to make changes to your financial disclosure, please include your updated statement in your cover letter. Guidelines for resubmitting your figure files are available below the reviewer comments at the end of this letter.*

*If applicable, we recommend that you deposit your laboratory protocols in protocols.io to enhance the reproducibility of your results. Protocols.io assigns your protocol its own identifier (DOI) so that it can be cited independently in the future. For instructions see:* [*http://journals.plos.org/plosone/s/submission-guidelines#loc-laboratory-protocols*](https://nam02.safelinks.protection.outlook.com/?url=http%3A%2F%2Fjournals.plos.org%2Fplosone%2Fs%2Fsubmission-guidelines%23loc-laboratory-protocols&data=04%7C01%7Cjonathan.kominsky%40rutgers.edu%7Cae0013ea03ac4e0744d508d8a757431d%7Cb92d2b234d35447093ff69aca6632ffe%7C1%7C0%7C637443340548602074%7CUnknown%7CTWFpbGZsb3d8eyJWIjoiMC4wLjAwMDAiLCJQIjoiV2luMzIiLCJBTiI6Ik1haWwiLCJXVCI6Mn0%3D%7C1000&sdata=Ahp9SZXDwZ6dTEyDqozeLlsaZP1n81Q6Gm8Z%2Bbbk3zM%3D&reserved=0)

*We look forward to receiving your revised manuscript.*

*Kind regards,*

*Hanna Marno*

*Academic Editor*

*PLOS ON****E***

**Reviewer #1:**

*The authors examined whether preschoolers make systematic predictions about the insides of self-propelled objects. The paper is concise and clear and offers an interesting method for bridging two related lines of research that could not be directly compared before.

One comment relevant to present paper:

• It would be helpful to have adult data. The paper implies that the mature response pattern is systematic selection of the biological insides. This is implied by the developmental story wherein young children have ‘abstract’ expectations about insides of self-propelled furry objects but then acquire more specific biological understandings with age. But the self-propelled object looks artificial and engaged in simplistic movements that imply a toy rather than an animal. If adults systematically predict mechanical insides then that seriously limits the interpretation of the study. In particular, it may be that the developmental trajectory with these stimuli is a biological to mechanical switch: Infants make biological attributions, adults make mechanical attributions, and 4-year-olds are in the early-states of that switch. Though this limitation could also be present in the infant work, it is also highly plausible that infants lack requisite knowledge about which kinds of colors, materials (fur/feathers), and movements are commonly artificially reproduced. So, it is possible that infants are making biological attributions. In lieu of adult participants, it would be helpful if this possibility was addressed in the General Discussion.*

We thank this reviewer, and Reviewer 2, for bringing this concern to the fore. We have expanded our discussion to more fully explore the problem of what children’s responses mean and whether or under what circumstances they treat these entities as mechanical toys. In fact we go further than that and incorporate the possibility that they do (accurately) view these entities as puppets, that is, as covering a smaller independent agent. These changes can be found throughout the General Discussion.

*A few thoughts for authors to consider for future directions:

• Authors might consider a separate project investigating exactly this question of animal / artifact attributions; It could be really interesting to see how expectations about the appearance and locomotion/behavior of animals develops. This would be particularly interesting cross-culturally. You may find that children continue to perceive the self-propelled object as an animal if they lack exposure to artifacts with bright colors and the ability to move (i.e., children’s toys in industrialized contexts). Whereas children with exposure to these kinds of artificial objects might begin to make mechanical inferences.

• In a future study, authors may consider a two-step procedure: First ask children whether they think the object is empty or not, and then ask what they think the insides are. For the ‘Inert’ condition: Presently, if a third of children expect no insides and two-thirds expect insides (but are 50/50 between biological and mechanical), their responses could resemble chance-responding overall when they are systematically expecting insides. For the ‘Self-propelled’ condition: Children that predict insides strongly (~80% of the time) but are 50/50 between biological and mechanical look only weakly different from chance when they might be very different from chance: My guess is you would find children selecting ‘has insides’ 50% of the time in the ‘Inert’ condition and 80% of the time in the ‘Self-propelled’ condition, which would better reflect the strength of their intuitions rather than the current results which look weaker that I bet children’s expectations really are.

• For future work, it may be worth exploring the addition of psychological behaviors, such as interacting contingently or pursuing goals.*

We thank the reviewer for these exciting suggestions for future work, which we now include in the General Discussion.

**Reviewer #2:**

*The manuscript addresses an important issue in conceptual development concerning how children represent the insides of animate and inanimate objects. The authors clearly identify a gap in the literature and ask whether children possess concrete biological expectations about the animals’ insides. The study replicated previous findings with infants and preschool-age children and the authors conclude that 4-year-olds have abstract expectations about the insides of self-propelled objects, but don’t distinguish between mechanical and biological insides. While I acknowledge that this is an interesting study, I have some major concerns that I mention below:*

*1. Setoh, et. al (2013) and others (Taborda-Osorio & Cheries, 2018) have proposed that infants infer the presence of internal material properties whenever an object exhibits two features at once: it is self-propelled and agentive. “Agentivity” refers to those features in the object’s behavior that are diagnostic of intentionality; such as, contingent behavior, eyes or fur. In the introduction section this distinction it’s unclear and the authors seem to imply more than once that the key diagnostic feature is only the self-propelled motion. Accordingly, they try to replicate one of the experiments of the Setoh, et. al.’ study without giving much explanation as to why the presence of both self-propelled behavior and fur covering the object would allow children in principle to infer internal material properties. I think this issue deserves further elaboration in both the introduction and the discussion section.*

We thank the reviewer for this suggestion, and have expanded our introduction and general discussion to elaborate the role of these additional features beyond self-propelled motion.

*2. The study compares the results of two tests, the self-propelled and the biological/mechanical intuition test. I find the results of both tests contradictory, in both cases children are supposed to represent an object as an “animal”, according to the results in the first test they don’t have biological expectations but in the second one they do have biological expectations, replicating the original findings. Why? The authors stress that in one test the objects are still while in the other are self-propelled, but again if both entities are represented like animals, we would expect a similar pattern of results. This issue should be brought out in the discussion section. For instance, are the insides of self-propelled objects less clearly identify with something biological?*

We have expanded our discussion of this significantly, see also our response to commnet (3) below.

*3. Related to the previous point, one possibility is that the children in the current study are not representing the self-propelled object like an animal like infants do. Unlike 8-month-old infants, 4-year-olds have a great deal of knowledge about how a typical animal looks like and they also have experience with mechanical toys. A self-propelled fur-covered object may be something rather atypical as an animal exemplar, in between a mechanical toy and a real animal (also notice that mechanical fur-covered toys are pretty common today). Something striking in the pattern of results is that in the self-propelled condition children have a (significant?) preference for mechanical insides compared to empty insides, but probably a non-significant difference between empty and biological insides. Thus, one possibility is that the children here represent the object like a mechanical toy and are biased to attribute mechanical insides which could be more coherent with their daily-life experience.*

This point is very convergent with the issues raised by Reviewer 1. Re-summarizing our response here, we have expanded our discussion to consider the role of a child’s idiosyncratic experience in their understanding of these objects. Thank you for this suggestion.

*4. I believe the hypothesis for biological expectations is ruled out too soon. What would happen if children witness a more realistic animate object (contingent behavior, eyes?)? would they still be agnostic as to what type of insides they have? If under these conditions they still don’t show any preference for one or the other I think the conclusion would be better supported. I believe the study would benefit a great deal from a second experiment with stimuli displaying more agentive features. I insist that the infants and children’s inference about the presence of insides is a matter of both self-propelled motion and also agentive features (see also Opfer & Siegler, 2004). Stronger evidence for agentive behavior may lead children to prefer the biological insides in the test. If this is the case, the comparison across both experiments would be very informative. In its current form, the study is mainly about children’s intuitions about the insides of self-propelled objects rather than the insides of animate agents.*

As mentioned in our reply to R1, we now discuss the possibility that some children may regard these as animals while others do not, but we predict that if we made these entities look more like animals, children would be more likely to pick the biological insides, or at a minimum reject the mechanical insides. We also thank the author for reminding us about Opfer & Siegler (2004), which we now cite.

**Reviewer #3:**

*In "There's something inside": Children's intuitions about animate agents, the authors tested whether 4-year-olds have intuitions about whether or not self-propelling vs inert objects have insides, and whether they have intuitions about the specifics of the insides. They tested this by showing novel objects (fur-covered boxes) that were either self-propelling or inert, and asked participants to choose between “X-rays” of the insides (empty, mechanical, or biological). The 4-year-olds were more likely to say that self-propelled objects are non-empty, but did not have an intuitions regarding the specifics of the insides of unknown objects.

The paper is well-written and a pleasure to read. The introduction clearly lays out the current knowledge and the question at hand, the design is straight-forward, and the authors have done a great job at moving the study online to test approximately 1/3 of the remaining participants after the pandemic hit. The authors have pre-registered their study, conducted a power analysis beforehand to determine the number of participants, and made their materials available, all of which is appreciated. I do still have a few questions and comments.*

We thank the reviewer for these very positive comments!

*Main comments:

My first comment pertains to the results of RQ1. Although you did a power analysis and found a statistically significant difference (p=.046), I wonder whether the result is as strong as the discussion section would make us believe. Due to publication bias, the estimated effect size used for the power analysis is often larger than the true effect size. I would be curious to know whether the authors have calculated the true effect size for this finding and whether that effect size reasonably warrants their conclusions? The authors’ claims are quite strong for a result that does not seem to have a convincing large effect size. Moreover, I would encourage the authors to calculate the Bayes Factor to determine whether there truly is more evidence for the alternative hypothesis than for the null hypothesis. A Bayesian chi-square analysis can be conducted in JASP, and the authors could even use the responses from the inert condition as the null hypothesis. My worries on this matter stem from the ideas regarding the uncanny p-mountain (see http://www.the100.ci/2018/02/15/the-uncanny-mountain-p-values-between-01-and-10-are-still-a-problem/) and the relatively low (only a third) “empty”-responses for inert objects.*

First, with regard to the strength of our claims, we have heavily revised the General Discussion to more fully consider what these results do and do not allow us to conclude, and what future work will be needed to address. (Please also see response to Reviewers 1 and 2.) We are now clearer that this paper reports an initial investigation, not a definitive conclusion.

With regard to the uncanny p-mountain, we point out that the intended goal of preregistration is to allow such results to be taken at face value, rather than be the result of this kind of publication bias. Given our estimated effect size in our power analysis, this result is entirely in the range of our expected effects.

Finally, we do not believe that the Bayesian chi-square analysis is appropriate to these data. To be clear, what we report (and preregistered) is a chi-square goodness-of-fit analysis comparing two mixed binary logistic regression models, not a chi-square test for independence. We see this was unclear and now clarify this in the results section. As such, a Bayesian (or frequentist) chi-square test for independence would be an entirely different analytical approach. Such an analysis would also not account for the fact that item (inert versus self-propelled) is a within-subjects factor. For these reasons we originally decided to use a binary logistic mixed-model analysis, and that is also what we based our power analysis on. We therefore believe it would be difficult to draw meaningful conclusions from such an analysis without redesigning the experiment.

*My second comment concerns the conclusions and discussion section. Although there is some previous literature that indicates that children may have causal beliefs about the insides of an objects and its ability to self-propel, these beliefs were not tested in this study. I think this warrants a more nuanced discussion differentiating between about what can be concluded directly from the study itself and what potential implications may be considered given previous findings. I recognise that the authors have already nuanced their conclusions in part, but I feel there is still too much of a jump from the measurements of their design to the way the results are discussed. In addition to a clearer distinction, a few sentences on how the parameters (design, measures, age groups, etc.) of the previous studies (that found causal beliefs relating to insides and agency) relates to the authors’ study might help the reader to better understand in which ways the earlier findings can be generalised to this study and where potential differences might be.*

Thank you for this point.We have tempered our conclusions substantially in line with the comments of all three reviewers. We now discuss potential differences due to age as well as the level of industrialization and exposure to self-propelled artifacts in the child’s local environment

*Minor comments:

How did you deal with potential parental interference during the online version of the study?*

We have added a footnote to the methods explaining that we were prepared to make exclusions on the basis of overt parental interference, but did not observe any such interference in this sample.

*The data on OSF is only available after requesting access (no problems accessing the pre-registration). In the end I managed to find a way to the data via the pre-registration, but you might want to have another look at the settings or mention the requirement for requesting access.*

We have double-checked our access settings on OSF to ensure that reviewers can access all of the relevant materials and information.

*References: It is difficult to see that (14) refers to the same paper as Simons & Keil (1995) without having to scroll back and forth. Obviously, this is due to the referencing system of the journal and not the authors’ doing. However, maybe the authors can do something to make it easier on the reader (e.g. write such situations as: Simons and Keil (1995) were exceptional [...] could readily identify (14).)? I don’t know whether PLOS ONE allows the authors to do this and it is only a very minor point.*

We agree that it is difficult to track this style of reference. We have added the reference number to each mention of a paper in text per this recommendation.
