# Supplementary material for: “There’s something inside”: Children’s intuitions about animate agents
Source: PLoS One. 2021 May 19;16(5):e0251081. doi: 10.1371/journal.pone.0251081 (PMC8133430; doi:10.1371/journal.pone.0251081)
Supplement: Attachment — Submitted filename: R2R.PONE-D-20-34496.docx [file pone.0251081.s002.docx]

April 12, 2021

Dear Dr. Marno,

We are pleased to have the chance to revise our short report, “‘There’s something inside’: Children’s intuitions about animate agents”. We are appreciative of the chance to have revised the manuscript and glad that the reviewers were generally satisfied by our previous set of revisions. Reviewer 3 has raised two final comments which we address below and in the revised manuscript:

*Although I take the point about preregistration as a means of showing that the analysis was planned and not a lucky shot, I do not think that preregistering their power analysis (based on previous literature) necessarily means it is unaffected by publication biases. It is not unlikely that the calculated sample size is based on an overestimated effect size. I suggest the authors report the observed effect sizes to provide better estimates for future studies to calculate power.*

Thank you for this suggestion. We have added effect sizes for all significant findings for which effect sizes can be computed.

*Regarding the discussion, much of it is focused on biological vs mechanical and it offers an appropriately nuanced view now. However, I think it would be good if the authors also spend a few words in the discussion on the number of “empty” responses related to RQ1. Although the difference with self-propelled was significant, the number of empty responses for inert items was only 31/92. What consequences does it have for the validity of the research question if children are not very likely to consider any object empty? What does it mean for agency assessment that in 61 cases, the child attributed insides to an inert object as well?*

Thank you -- we are glad you find the revision more nuanced! We have added a paragraph on pg. 15 that directly addresses your concern regarding interpretation of the “empty” responses. In short, we believe that the behavior of an ‘inert’ object does not give positive evidence that an object is empty. Rather, it gives no evidence at all about an object’s internal properties, and so children respond randomly.

Thank you very much for your continued consideration.

Sincerely,

Jonathan F. Kominsky, Patrick Shafto, and Elizabeth Bonawitz
